# The effects of exercise on microRNA expression profiling in adipose tissue macrophages of mice

**DOI:** 10.3389/fimmu.2024.1412621

**Published:** 2024-08-19

**Authors:** Fei Qin, Wenbai Huang, Chaoyi Qu, Lina Zhao, Yunyu Du, Tianyu Zhao, Yiwei Feng, Jiexiu Zhao

**Affiliations:** ^1^ School of Physical Education, Jinan University, Guangzhou, China; ^2^ Exercise Biological Center, China Institute of Sport Science, Beijing, China; ^3^ Guangdong Provincial Key Laboratory of Speed Capability Research, Su Bingtian Center for Speed Research and Training, Jinan University, Guangzhou, China; ^4^ Physical Education College, Hebei Normal University, Shijiazhuang, China; ^5^ School of Physical Education, Shanxi Datong University, Datong, China; ^6^ Athletic Sports Research Lab, Beijing Institute of Sports Science, Beijing, China

**Keywords:** exercise, inflammation, metabolic syndrome, adipose tissue macrophages, bioinformatics analysis, microRNA

## Abstract

**Background:**

Exercise is recognized for its broad health benefits, influencing various physiological processes, including the behavior of adipose tissue macrophages (ATMs). While existing studies mainly associate ATM activity with obesity and metabolic syndrome, our study explores the impact of aerobic exercise on ATM microRNA expression profiling in a non-obese context, highlighting its general health-promoting mechanisms.

**Methods:**

Sixty male C57BL/6 mice were randomly assigned to either a sedentary (S) or an exercise (E) group. The S group remained inactive, while the E group underwent a one-week treadmill adaptation, followed by an 8-week aerobic treadmill exercise protocol (60 min/day, 5 days/week, at 65%-75% VO_2max_). Post-training, glucose tolerance and the serum lipid levels were measured in mice subjected to both exercise and non-exercise conditions. ATMs harvested from visceral adipose tissues were analyzed and sorted using flow cytometer. To further investigate the effects of exercise in ATMs at the molecular level, miRNA microarray analysis was performed, followed by bioinformatic analysis.

**Results:**

The 8-week regimen of moderate-intensity aerobic exercise ameliorated glucolipid metabolism and fostered a dynamic shift toward an M2 macrophage phenotype in the adipose tissue, independent of obesity. A total of 62 differentially expressed miRNAs were identified in ATMs of mice post-exercise. Notably, six miRNAs (miR-212-5p, miR-511-5p, miR-7b-5p, miR-142-3p, miR-1894-3p, and miR-31-5p) as well as their target gene were consistently altered and associated with macrophage polarization and metabolic regulation.

**Conclusion:**

Our findings broaden the understanding of how exercise regulates ATM functions through significant changes in microRNA profiles, emphasizing its potential to enhance health and prevent chronic conditions. This study supports the application of aerobic exercise for its preventive effects on chronic diseases and underscores the importance of microRNA profiling in understanding the immune-modulatory impacts of exercise.

## 1 Introduction

While metabolic syndrome, a complex condition characterized by obesity, insulin resistance, and dyslipidemia, is traditionally linked to chronic low-grade inflammation and adverse health outcomes (1), current research increasingly explores broader applications of exercise beyond disease mitigation. Adipose tissue macrophages (ATMs), which can constitute up to 50% of the cells in adipose tissue of obese mice (2), play a pivotal role in both metabolic and immune regulation of adipose tissue. This is particularly evident not only in pathological states but also under baseline health conditions (3–5). Although much of the existing research on ATMs has centered on their functions within the pathological states of obesity and metabolic syndrome, the shift in focus acknowledges that the benefits of exercise transcend the confines of obesity and metabolic syndrome, suggesting preventative and health-promoting effects that are universally relevant.

Exercise training is recognized for its capacity to ameliorate various metabolic dysfunctions (6–9) and to alleviate systemic inflammation (10). Specifically, long-term aerobic exercise training (8-16 weeks) induces anti-inflammatory shifts in macrophage phenotypes from pro-inflammatory M1 to anti-inflammatory M2, affecting both humans and rodents (11–13). However, the exact molecular mechanisms, particularly the role of microRNAs (miRNAs) in these process, induced by exercise intervention, remains elusive outside of disease contexts.

MicroRNAs are small non-coding RNAs composed of 19–25 nucleotides, which bind to mRNA, leading to translational arrest and mRNA degradation (14). These mechanisms allow miRNAs to downregulate the protein expression of their targeted mRNAs, thereby serving as regulators of mRNA expression and translational efficiency across most cell types (3). Over the past decade, miRNAs have been deemed as key regulators of metabolic homoeostasis. The development of metabolic syndrome in the major tissues, such as liver, skeletal and heart muscle as well as adipose tissue, are all affected by miRNAs (15). Additionally, miRNAs have been shown to mediate the effects of exercise intervention, improving the conditions such as obesity (16), cardiovascular diseases (17), diabetes (16). Consequently, the regulation of miRNAs may represent a crucial area of research, potentially linking exercise to regulation of metabolism and inflammation in ATMs in non-pathological states.

Exercise, by its nature, is a complex physiological process that impacts multiple systems and signaling pathways in the body simultaneously. Due to this complexity, primary cell isolated from exercised subjects is considered more effective than simplified *in vitro* models of exercise. These cells better capture the multifaceted biological responses to physical activity, offering a holistic and physiologically relevant representation of the body’s interconnected cellular and molecular mechanisms activated by exercise. To delve into the precise mechanisms of the ATMs in response to exercise, we utilized exercise-induced primary ATMs and miRNAs microarray to investigate the effects of an 8-week aerobic exercise training on ATMs. Following the exercise regimen, ATMs were sorted using flow cytometer and subsequently analyzed through bioinformatics analysis to identify changes in miRNA expression. Our study reveals that aerobic exercise leads to significant changes in the expression of miRNAs and corresponding genes in ATMs, which are linked to improved metabolic health even in non-pathological conditions. These findings enrich our understanding of how exercise can fundamentally modulate immune and metabolic responses through miRNA-mediated pathways in ATMs, offering new insights into the broad applications of physical activity. By elucidating these mechanisms, our research opens potential avenues for developing strategies to manage inflammation and design exercise programs that optimize health outcomes across various populations, highlighting the preventive and therapeutic potential of exercise in enhancing general well-being.

## 2 Materials and methods

### 2.1 Animals

Sixty 6-week- old male C57BL/6 mice were acquired from the Beijing Vital River Laboratory Animal Technology Co., Ltd. All animals were randomly divided into either sedentary (S) group (n = 30) or exercise (E) group (n = 30) after one-week acclimatization, and were housed individually in ventilated caging system under a 12:12-hour light-dark cycle at 23 **±** 2°C and 45%–55% humidity. The mice maintaining chow (11.85% fat, 65.08% carbohydrate, 23.07% protein) were purchased from Beijing Keao Xieli Feed co., ltd. The animals were allowed free access to food and water. All protocols of the experiment were approved and conducted in accordance with the guidelines set by the Animal Ethical Committee of the China Institute of Sports Science, Beijing, China.

### 2.2 Exercise program

The aerobic exercise protocol for the E group included a one-week treadmill adaptation phase, followed by 8 weeks of moderate aerobic treadmill exercise training (18). The adaptation phase involved 20 minutes of daily treadmill (DSPT-202, China) running at 65%-75% maximal oxygen uptake (VO_2max_) for 5 days per week, to acclimate the mice to the exercise routine. Each session in the main exercise regimen lasted 60 minutes at an intensity of 65%-75% VO_2max_, incorporating a 5-minute warm-up and a 5-minute cooldown at a reduced intensity of 45%-55% VO_2max_. This exercise regimen was conducted 5 days a week for 8 weeks. To ensure optimal exercise intensity and adapt to the mice’s improving fitness levels, the treadmill running speed was set initially and then adjusted bi-weekly based on the VO_2max_ assessments. During this period, the mice in the S group remained inactive. No significant difference in weight was observed between the two groups of mice post-training (S vs. E: 26.38 **±** 1.06 vs 25.5 **±** 0.93 g, p = 0.1006).

### 2.3 Glucose tolerance test and serum lipid levels

Following a 16-hour fast, mice was subjected to intraperitoneal glucose tolerance tests (IPGTT). Briefly, blood was collected from a small incision in the tip of the tail at 0 min (fasting blood glucose) and subsequently 15, 30, 60, 90 and 120 min following an intraperitoneal injection of glucose (2 g/kg body weight). Blood glucose levels were measured with a blood glucometer (Accu-Check Active, Roche). Total cholesterol (T-CHO), triglyceride (TG), low-density lipoprotein cholesterol (LDL-C), and high-density lipoprotein cholesterol (HDL-C) were measured using T-CHO (Cat# A111-1-1), TG (Cat# A110-1-1), LDL-C (Cat# A113-1-1), and HDL-C (Cat# A112-1-1) assay kits (Nanjing Jiancheng Bioengineering Institute, Jiangsu, China) and an enzyme-linked analyser (MultiskanAsc, Thermo, USA). The TNF-α (Cat# F12044) and IL-10 (Cat# F04098) content in the serum were measured by enzyme-linked immunosorbent assay kits (Yanjin biological, China) according to the manufacturer’s instructions. Optical density was recorded at 450 nm with an enzyme-linked analyser (MultiskanAsc, Thermo, USA).

### 2.4 Isolation of stromal cells from visceral adipose tissue

Visceral adipose tissues (VATs) of mice were isolated using a sterile technique and mechanically minced. The mince VATs were then digested using collagenase II (2 mg/ml, Sigma-Aldrich, Cat#C6885) for 30min at 37°C. After digestion, the bottom layer of the cell slurry, followed by the adipocyte-containing upper layer, was transferred onto the 100-mm nylon filter prewet with PBS using a transfer pipette. The filter was installed to a centrifuge tube and then centrifuged at 1000g for 10 min at 4°C. After centrifugation, primary adipocytes formed a white layer at the top, while the stromal vascular cell (SVC) settled as a red/white pellet on the bottom of the tube. The pellet was then incubated with red blood cell lysis buffer to remove any erythrocytes. Finally, the cell pellets were resuspended in FACS buffer for further analysis.

### 2.5 Adipose tissue macrophage isolation and fluorescence-activated cell sorting

SVCs isolated from VATs were resuspended in FACS buffer to achieve a final concentration of 1×10^7 SVCs/ml. Subsequently, 1 mg of Fc-block (anti-CD16/32, eBioscience, Cat# 14-0161-81) was added, and the suspension was incubated on ice for 10 minutes. SVC single-cell suspensions were then incubated with fluorescence-tagged antibodies against CD11b (eBioscience, Cat# 53-0112-82) and F4/80 (eBioscience, Cat# 25-4801-82). Lastly, CD11b+ F4/80+ cell population were sorted as the ATMs using BDFACS Aria II flow cytometer (BD Biosciences) for further analysis (3).

### 2.6 Flow cytometry analysis

Surface receptor of CD11b, F4/80 and CD11c (eBioscience, Cat# 17-0114-82) labelling, cells were incubated with the marker for 30 min at 4**°**C in the dark. Cells were fixated and permeabilized with BD Cytofix/Cytoperm™ Fixation/Permeabilization Kit (Cat#554714). After, intracellular receptor of CD206 (Biolegend, Cat# 141706) labelling, cells were incubated with the marker for 30 min at 4**°**C in the dark, and wash and resuspend in FACS buffer. Data acquisition was performed using the BDFACS Aria II flow cytometer (BD Biosciences). M1 and M2 macrophages were defined as F4/80+ CD11c+ CD206 - cells and F4/80 + CD11c - CD206 + cells, respectively (3). Data analysis was carried out using FlowJo software 10.0.5 (Tree Star, Ashland, OR).

### 2.7 miRNA microarray

Total RNA was extracted from FACS-sorted ATMs using Trizol reagent (Invitrogen, Cat# 15596-026). Affymetrix miR 4.0 array analysis was conducted according to the manufacturer’s instructions. Briefly, 500ng of total RNA of each sample (n = 3) underwent a tailing reaction (FlashTag Biotin HSR RNA Labeling Kit, Affymetrix, Cat# 901911) and biotinylated. The miRNA profiling was performed using GeneChip ® miRNA 4.0 Array. After staining, the microarray chip was scanned using a GeneChip Scanner 3000 7G.

### 2.8 Bioinformatics analysis

Differentially expressed miRNAs were identified using the random variance model (RVM) t test. A threshold of 1.3-fold change (E versus S) and a P < 0.05 were set to distinguish between up- and down-regulated miRNAs. For gene expression pattern analysis, Cluster and Java TreeView software (version 3.0) were utilized. To predict the target genes of these miRNAs, we employed Target Scan Mouse V 7.1 and miRDB V5 databases, intersecting their results to enhance the reliability of the results.

Gene Ontology (GO) analysis was conducted to uncover the primary functions and biological processes of the differentially expressed genes. Fisher’s exact test was used to classify the GO categories, and a false discovery rate (FDR) was applied to correct the p-values. The lower the FDR, the lower the likelihood of error in interpreting the p-values.

Significant signaling pathways of the differential miRNA target genes were analyzed using the Kyoto Encyclopedia of Genes and Genomes (KEGG), Reactome, and BioCarta databases. Fisher’s exact test was employed for this analysis, considering p < 0.05 as statistically significant. The representative signal networks of miRNA-mRNA interactions were visualized using Cytoscape V3.7.1, providing a comprehensive graphical representation of the complex interactions between these molecules. To validate the key regulatory genes which associated with lipid metabolism, insulin resistance, experimental validation was conducted based on the differential gene signaling pathways identified in the KEGG analysis.

### 2.9 Validation of miRNAs with qRT-PCR

Total RNA of 8 mice in each group was extracted using Trizol (Invitrogen, USA, Cat# 15596-026) from ATMs according to the manufacturer’s protocol. qRT-PCR reactions were performed using ABI PRISM 7900HT Real-Time PCR System (Applied Biosystems, CA, USA). Relative quantification of miRNA expression was calculated using the 2-△△CT method. miRNA-specific qRT-PCR primers were shown in **Table 1**.

**Table 1:** RT-qPCR primer sequences.

Gene	Primer sequence (5’ to 3’)
miR-511-5p	GCAGATGCCTTTTGCTCT
miR-212-5p	GCAGACCTTGGCTCTAGAC
miR-1894-3p	GCAAGGGAGAGGGTGAAG
miR-142-3p	CGCAGTGTAGTGTTTCCT
miR-31-5p	GGCAAGATGCTGGCATAG
miR-7b-5p	CGCAGTGGAAGACTTGTGA

### 2.10 Statistical analysis

All data are presented as the mean ± standard deviation (SD). The Shapiro–Wilk test was detected for normality of the data distribution. Student’s T test was performed for comparison between the groups. miRNA levels in serum, as determined by qPCR, were normalized with log2 transformation. Statistical analysis was conducted using SPSS software (version 22.0, IBM SPSS Statistics, Chicago, IL). A p-value of < 0.05 was considered statistically significant.

## 3 Results

### 3.1 Effects of 8-week aerobic exercise on glucolipid metabolism and systemetic inflammatory profile

**Figure 1:**
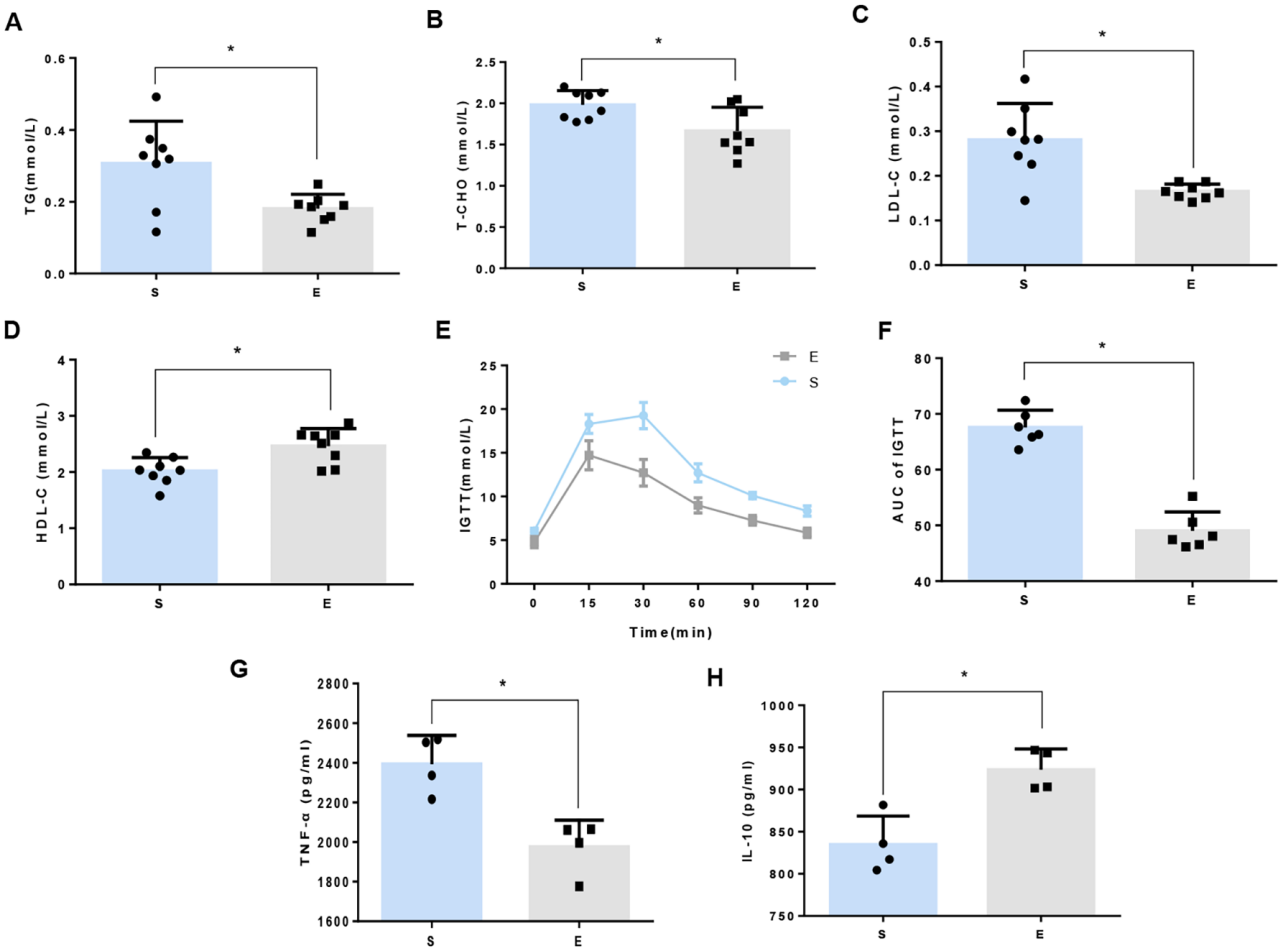
8-week aerobic exercise ameliorates disorders of glucose and lipid metabolism in mice. **(A)** Triglycerides (TG). **(B)** Total cholesterol (T-CHO). **(C)** Low density lipoprotein cholesterol (LDL-C). **(D)** High-density lipoprotein cholesterol (HDL-C). Data are presented as mean ± SD (n=8). **(E)** Intraperitoneal glucose tolerance tests (IPGTT). **(F)** Area under the curve (AUC) of IPGTT. *P<0.05 between groups. Data are presented as mean ± SD (n=6). **(G)** Tumor Necrosis Factor-alpha (TNF-α). **(H)** Interleukin-10 (IL-10). Data are presented as mean ± SD (n=4).

To determine the influence of 8-week aerobic exercise on glucolipid metabolism and systemetic inflammatory profile, we measured serum concentrations of TG, T-CHO, LDL-C, and HDL-C, glucose tolerance as well as inflammatory cytokines in mice. We observed significant reductions in TG, T-CHO, and LDL-C (**Figures 1A-C**), and a marked increase in HDL-C in exercise mice compared to sedentary counterparts (**Figure 1D**). Additionally, blood glucose profiles and the area under the curve (AUC) of IPGTT were considerably lower in exercise group (**Figures 1E, F**). A significant decrease of TNF-*α* and an increase of IL-10 in exercise mice were observed compared to sedentary counterparts (**Figures 1G, H**).

### 3.2 Effects of 8-week aerobic exercise on ATMs

**Figure 2:**
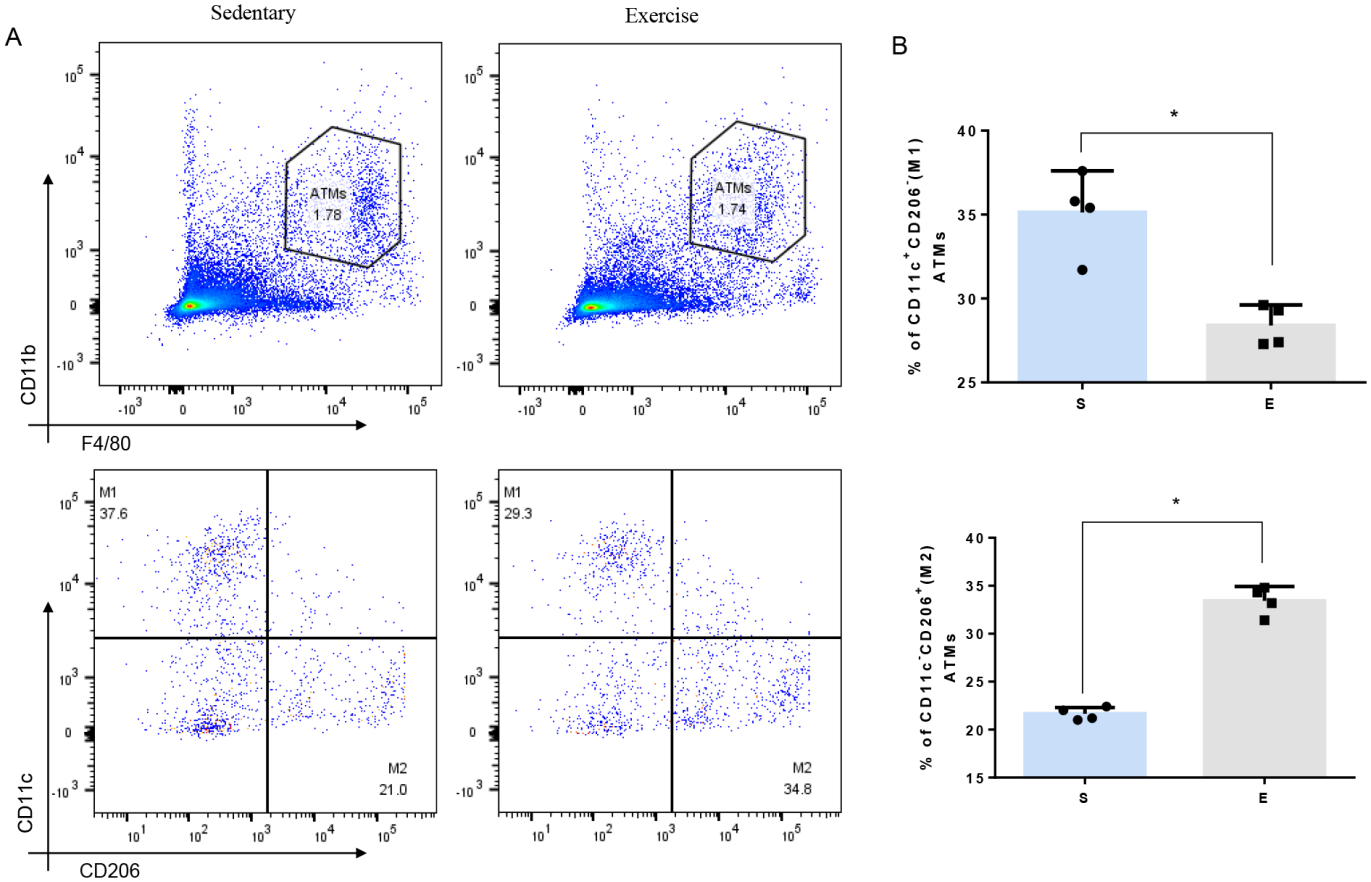
Effect of 8-week aerobic exercise on adipose tissue macrophages and subgroup of M1/M2. **(A)** Plot of total and M1/M2 macrophages in the adipose tissue of mice. **(B)** Quantitation of M1/M2 macrophages in the adipose tissue of mice (n=4, * P< 0.05 between groups).

Flow cytometry analysis was utilized to explore how 8-week aerobic exercise influences ATMs in mice. There was no statistical difference of the total number of ATMs between sedentary and exercise group (**Figure 2A**). However, a notable decrease in the proportion of M1-macrophages and an increase in M2- macrophages were observed post-exercise (**Figure 2B**). These results suggest that the 8-week exercise induces a shift toward an M2 macrophage phenotype in the adipose tissue, potentially suppressing the low-grade systemic inflammation. 

### 3.3 Identification of differentially expressed miRNAs in ATMs post 8-week aerobic exercise training

**Figure 3:**
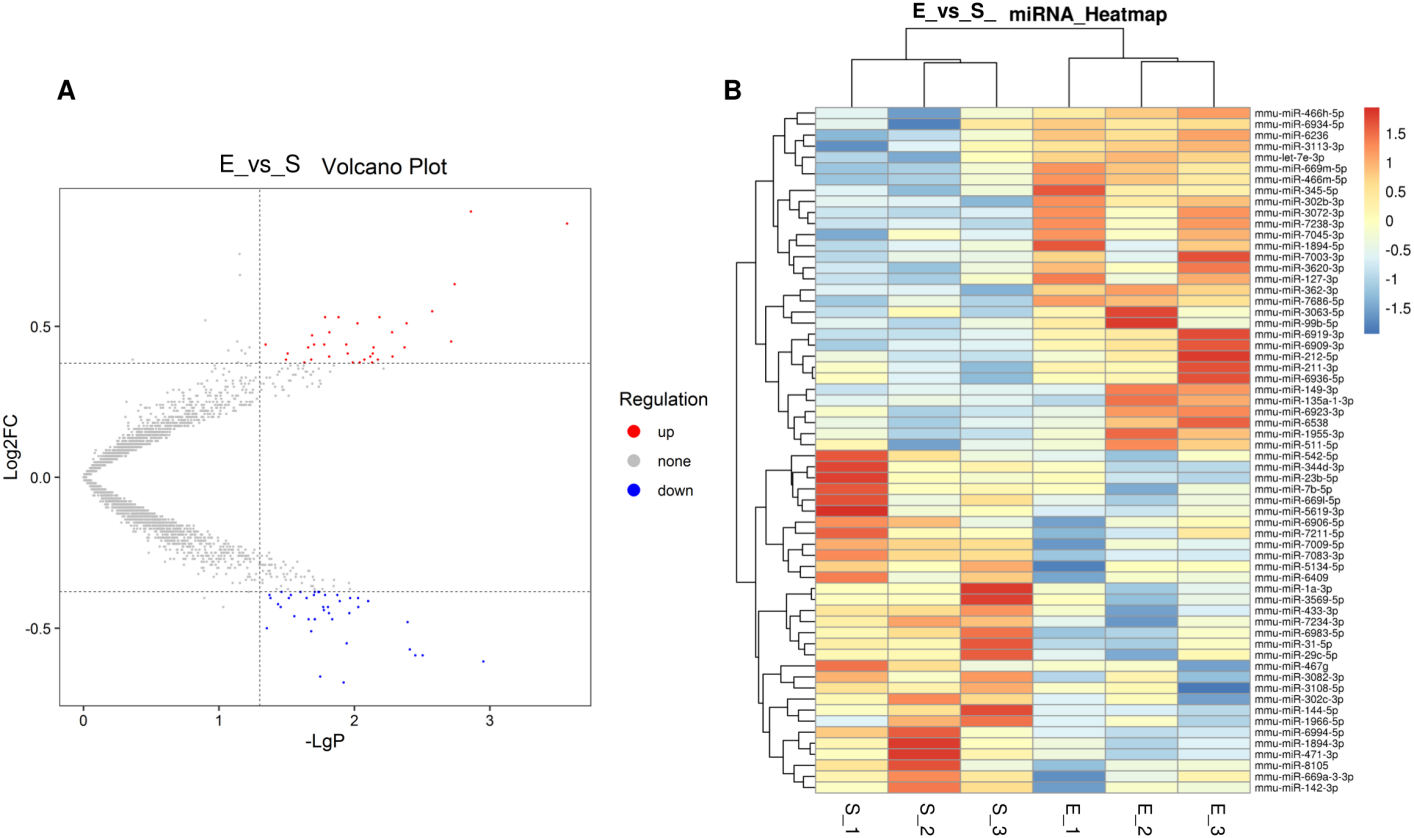
Hierarchical cluster analysis in ATMs triggered by 8-week aerobic exercise training. **(A)** Volcano of different miRNAs between in S group and E group. **(B)** Heatmap of different miRNAs between in S group and E group. 3 replicates per 20 mice per replicate.

Hierarchical cluster analysis was performed to investigate changes in gene expression patterns due to 8-week aerobic exercise training (**Figure 3A**). Microarray analysis identified 62 differentially expressed miRNAs following exercise regimen, of which 31 were downregulated and 31 were upregulated (FC > 1.3, P < 0.05) (**Figure 3B**). These results are detailed in **Supplementary Tables S1** and **S2**.

### 3.4 Bioinformatics-driven insights into miRNA target genes and associated biological pathways

**Figure 4:**
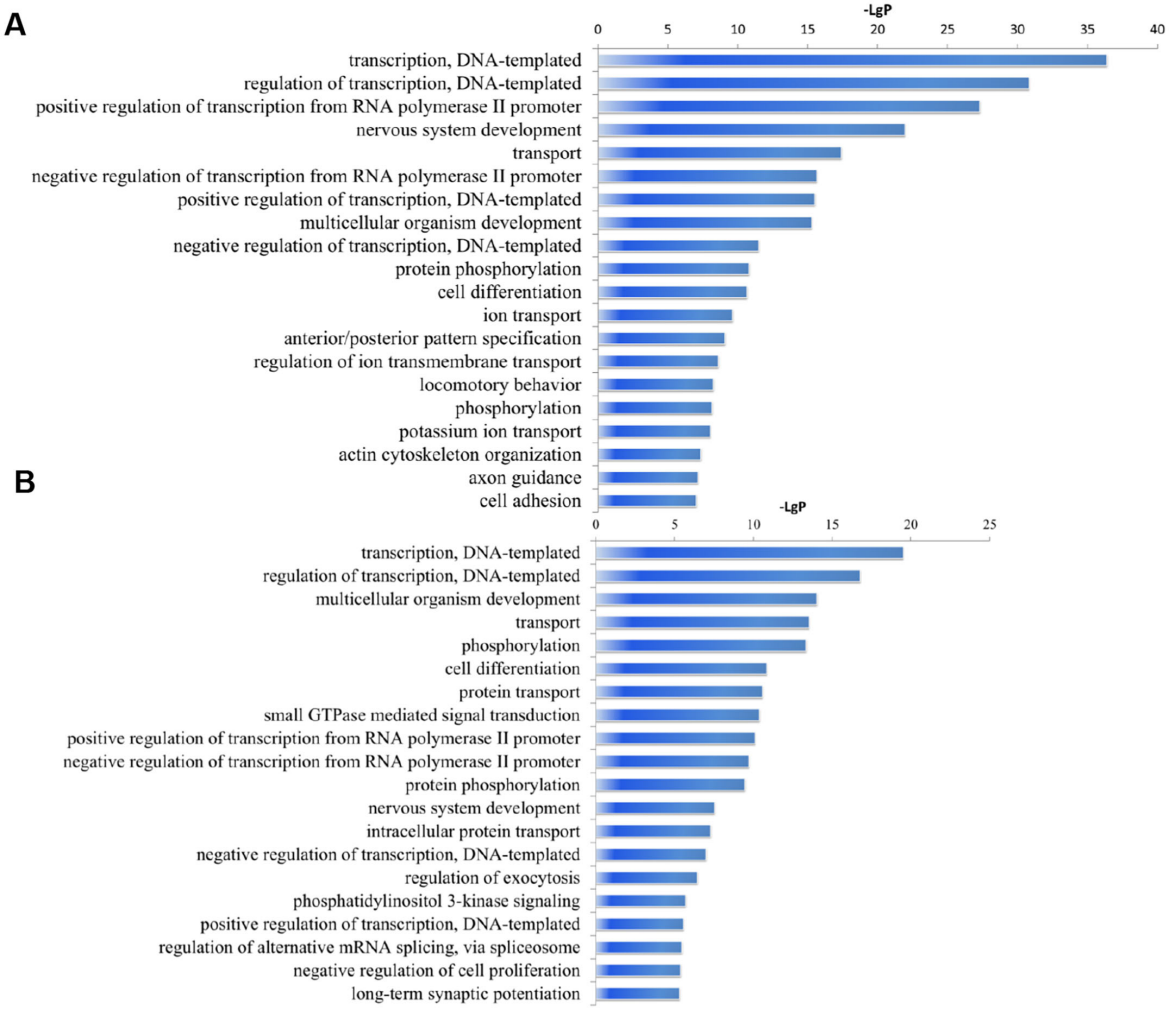
Significantly changes of Gene Ontology (biological processes) of miRNA target genes. **(A)** upregulated miRNAs. **(B)** downregulated miRNAs. The y-axis shows GO category and the x-axis shows−LgP where a larger−LgP indicates a smaller P-value. 3 replicates per 20 mice per replicate.

The Target Scan Mouse V7.1 and miRDB V5 databases were utilized for bioinformatic prediction to identify the potential target genes of the differentially expressed miRNAs. We identified 2864 target genes for the 31 downregulated miRNAs, and 4452 mRNA targets for the 31 upregulated miRNAs. To understand the primary biological processes associated with these genes, Gene Ontology (GO) analysis was performed. The top 20 upregulated GOs categories (biological process level) were transcription (DNA-templated), regulation of transcription (DNA-templated), positive regulation of transcription from RNA polymerase II promoter, nervous system development, transport and more (**Figure 4A**). The top 20 downregulated GOs categories include transcription (DNA-templated), regulation of transcription (DNA-templated), multicellular organism development, transport, phosphorylation, among others (**Figure 4B**).

**Figure 5:**
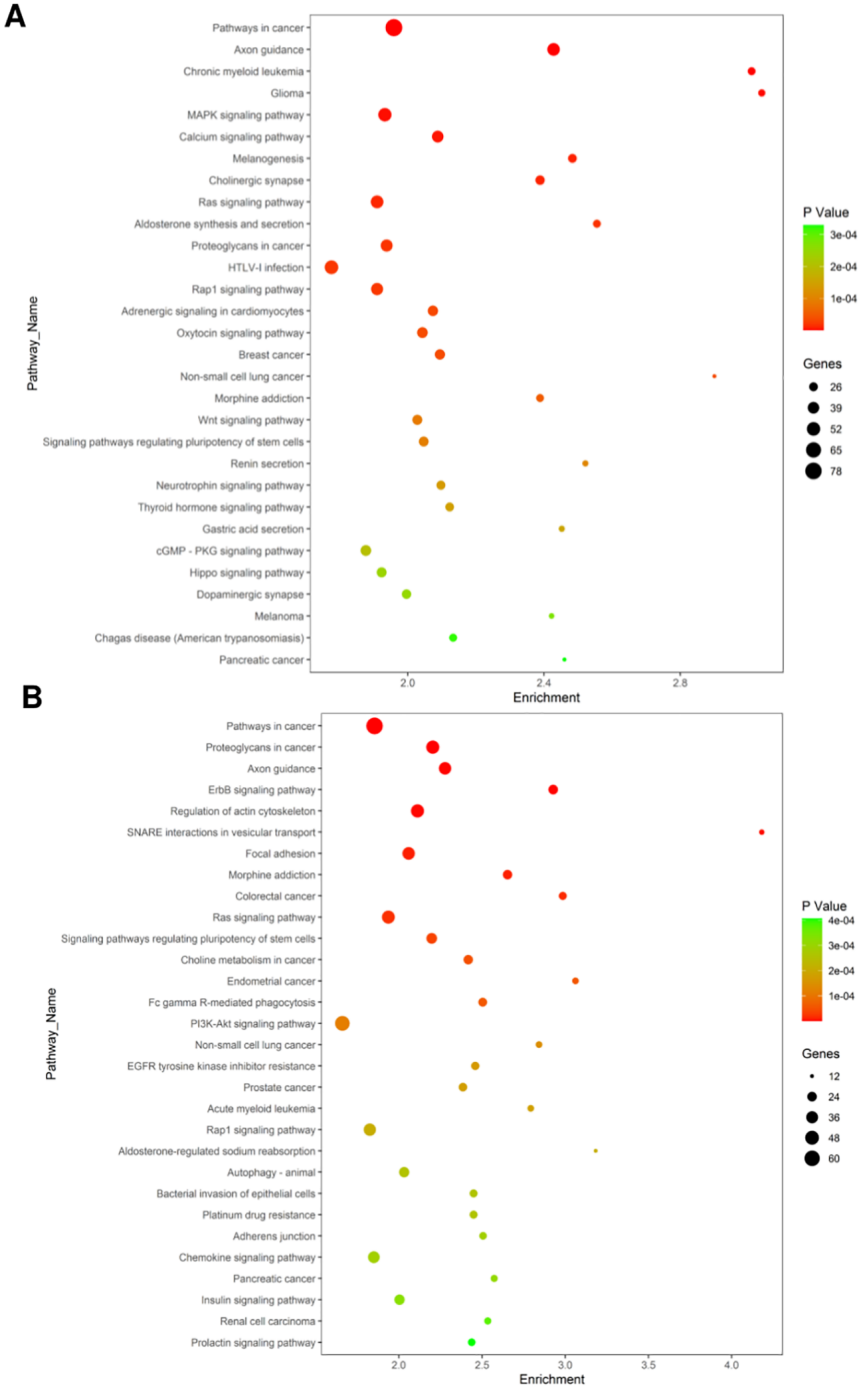
Significantly changes of Gene Ontology (biological processes) of miRNA target genes. **(A)** Biological pathways affected by upregulated miRNAs. **(B)** downregulated miRNAs. The X-axis indicates the number of enrichment gene in KEGG pathway category that mapping to the size of the dots. The color-coding indicates the elog10 (P-value). (For interpretation of the references to color in this figure legend, the reader is referred to the Web version of this article).

KEGG database analysis of the functions and interactions of differentially expressed genes revealed significant pathways influenced by the 8-week aerobic exercise regimen. The targets of upregulated miRNAs were involved in various pathways including those in cancer, axon guidance, chronic myeloid leukemia, glioma, MAPK signaling pathway, calcium signaling pathway, melanogenesis, cholinergic synapse, Ras signaling pathway, etc. (**Figure 5A**). The mRNA targets of the downregulated miRNAs participate in pathways related to cancer, proteoglycans in cancer, axon guidance, ErbB signaling pathway, regulation of actin cytoskeleton, SNARE interactions in vesicular transport, focal adhesion, morphine addiction, colorectal cancer, Ras signaling pathway and so on (**Figure 5B**).

### 3.5 miRNAs expression in exercise-induced ATMs is associated with metabolic syndrome

**Figure 6:**
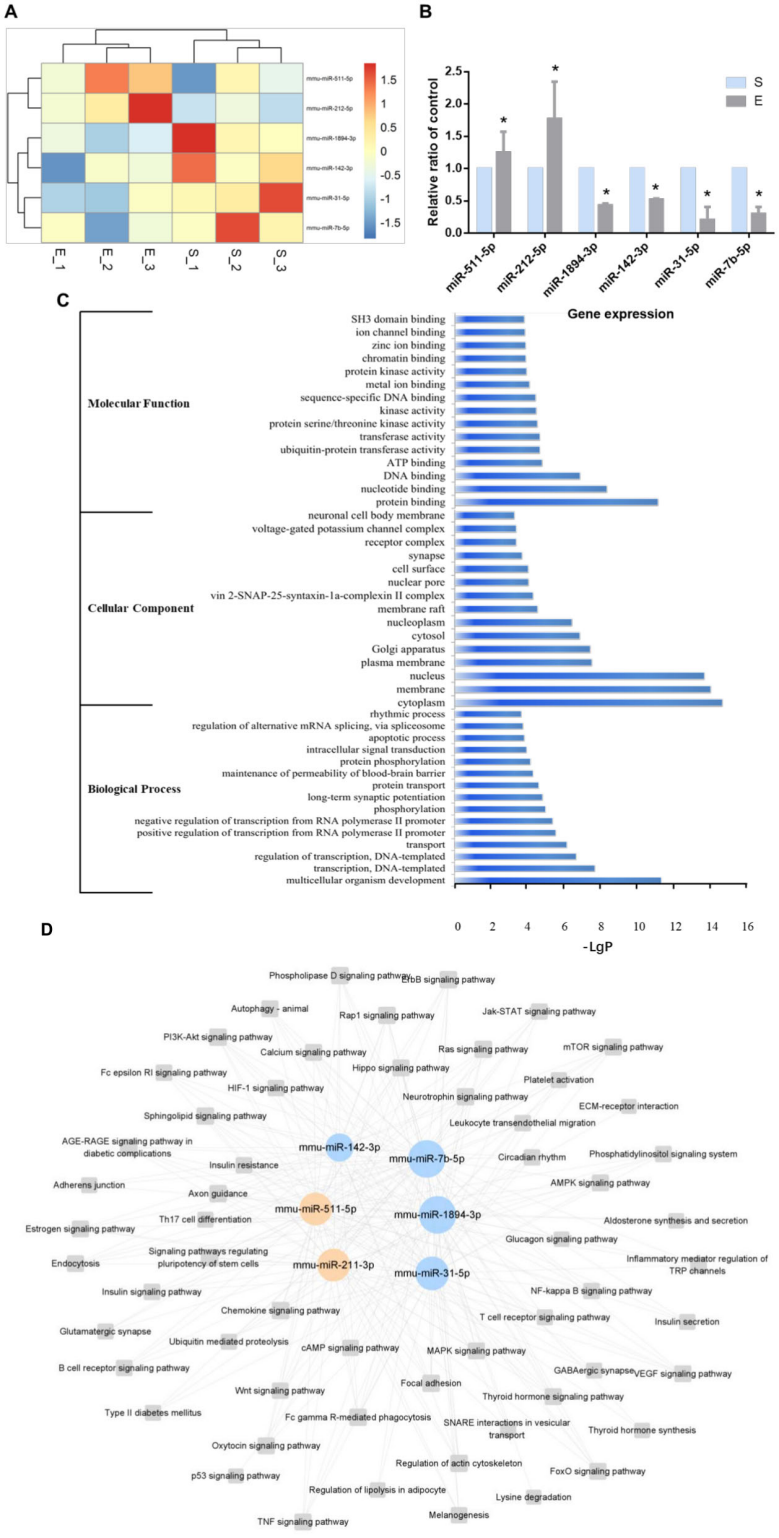
Six miRNAs post-exercise implicated in regulating metabolic syndrome. **(A)** Heatmap illustrating differentially expressed miRNAs associated with metabolic syndrome between the S group and the E group. **(B)** qRT-PCR confirmation of the 6 differentially expressed miRNAs post-exercise (n=6, *P-value < 0.05). **(C)** Top 15 GO enrichments of the in different functional groups (biological process, cellular component and molecular function). **(D)** A representative miRNA-pathway-network of the 6 miRNAs associated with metabolic syndrome (Grey box nodes represent the signaling pathway, orange cycle nodes represent upregulated genes, and blue cycle nodes represent downregulated genes). For a comprehensive view, refer to the detailed miRNA-pathway-network presented in **Supplementary Figure S1**.

Microarray analysis further identified 6 miRNAs (miR-212-5p, miR-511-5p, miR-7b-5p, miR-142-3p, miR-1894-3p, and miR-31-5p) among the 62 differentially expressed in ATMs post-exercise, all associated with aspects of macrophage polarization and metabolism regulation. KEGG pathways and GO analysis determined the upregulation of miR-212-5p and miR-511-5p as well as the downregulation of miR-7b-5p, miR-142-3p, miR-1894-3p and miR-31-5p (**Figure 6A**). These findings were corroborated by qRT-PCR, aligning with the initial microarray results (**Figure 6B**). The top 15 enrichments in each functional group (biological process, cellular component and molecular function) of these 6 miRNAs are depicted in **Figure 6**. In the biological processes (BP) group, genes were mainly enriched in multicellular organism development, transcription (DNA-templated), and regulation of transcription (DNA-templated) (**Figure 6C**). Within the cellular component (CC) group, enrichment was observed in the cytoplasm, membrane, and nucleus (**Figure 6C**). For the molecular function (MF) group, the main enrichments were in protein binding, nucleotide binding, and DNA binding (**Figure 6C**). KEGG pathway analysis was conducted to identify significant pathways associated with these miRNAs. The analysis revealed enrichment in key signaling pathways, including Wnt, PI3K-Akt, NF-kappa B, MAPK, mTOR, AMPK, and Insulin pathways, which are integral to macrophage polarization and metabolic regulation (**Figure 6D**). The miRNA-pathway-network, illustrating the relationships between these miRNAs and their target genes, is presented in **Supplementary Figure S1**.

**Figure 7:**
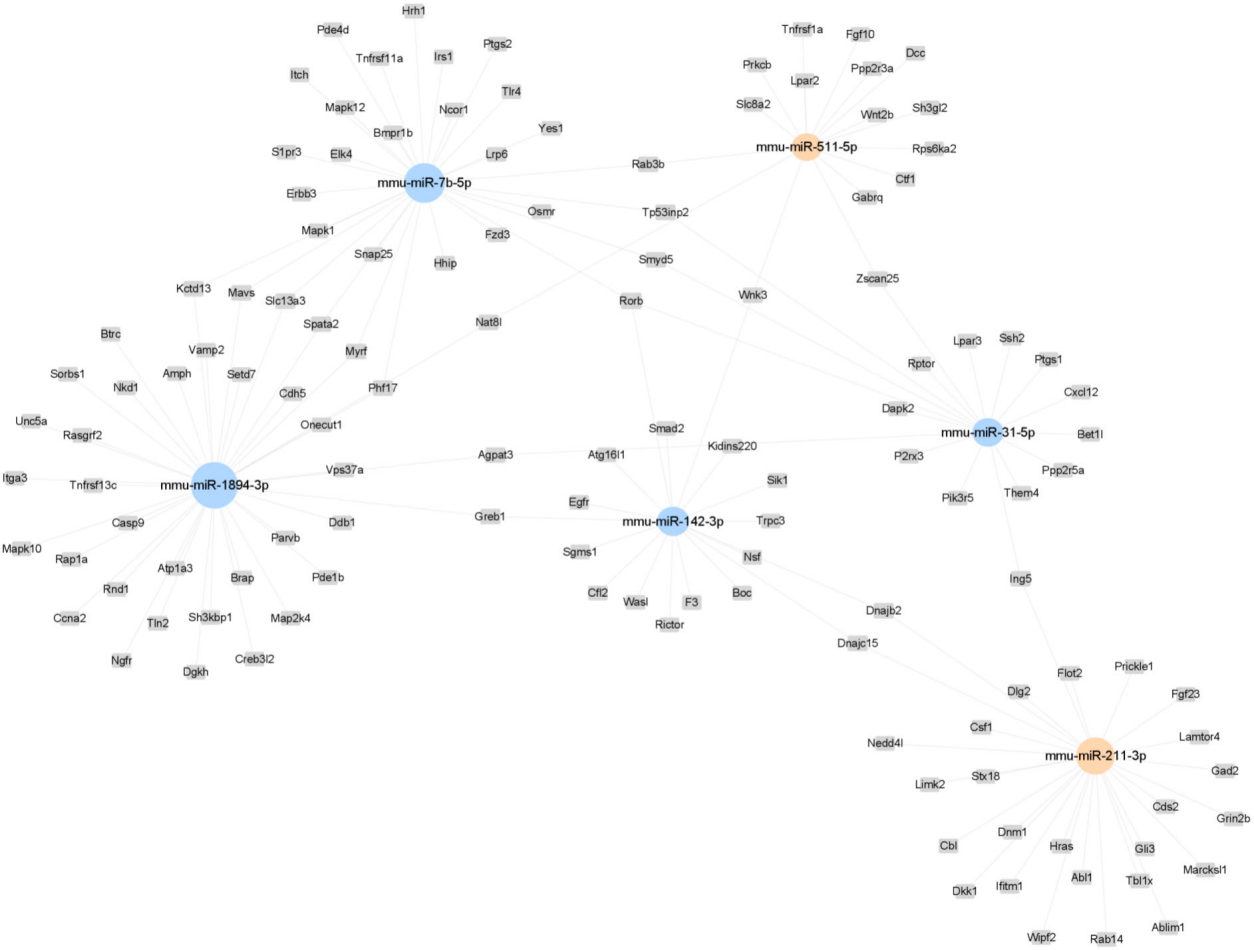
A representative miRNA-gene-network of the 6 miRNAs differentially expressed post-exercise. Grey box nodes represent the predicted target genes, orange cycle nodes represent upregulated genes, and blue cycle nodes represents downregulated genes. For a comprehensive view, refer to the detailed miRNA-gene-network presented in **Supplementary Figure S2**.

A miRNA-mRNA network was constructed to summarize the interaction between the selected miRNAs and their target genes. Rorb, Smyd5, and Tp53inp2 emerged as central to this network (**Figure 7**). Rorb and Tp53inp2 regulated by multiple miRNAs may be key molecules in metabolic regulation and inflammation mediated by ATM. Predictive analysis identified genes related to metabolism and inflammation, including Tnfrsf1a, Prkcb, Wnt2b, Fgf23, and Hras, as targets of upregulated miRNAs, and Mapk10, Nsf, Tp53inp2, Erbb3, and Irs1 as targets of downregulated miRNAs (**Table 2**).

**Table 2:** The target genes of miRNA differential expression.

miRNA	Regulation	Fold change	P value	Target gene
miR-511-5p	Up	1.444	0.013076	Tnfrsf1a, Prkcb, Wnt2b
miR-212-5p	Up	1.357	0.016655	Fgf23, Hras
miR-1894-3p	Down	1.366	0.015374	Mapk10
miR-142-3p	Down	1.301	0.018189	Nsf
miR-31-5p	Down	1.505	0.003129	Tp53inp2
miR-7b-5p	Down	1.385	0.019572	Erbb3, Irs1

## 4 Discussion

ATMs are closely connected to metabolic health, involved in regulating metabolic processes like glucose metabolism, insulin sensitivity, and lipid balance. Physical training has been demonstrated to improve these metabolic conditions, but the precise molecular pathways involved, particularly the role of miRNAs in mediating these effects, remain incompletely understood. To address this gap, our study used primary ATMs isolated from mice post-exercise, analyzing changes in gene and miRNA expression through microarray techniques after an 8-week aerobic exercise regimen. We identified 62 differentially expressed miRNAs in the ATMs, with six of these miRNAs showing consistent patterns linked to metabolic health improvements. These findings underscore the potential of aerobic exercise to beneficially modulate ATM function, even in the absence of metabolic disease. This research enhances our understanding of how physical activity influences ATMs at a molecular level in healthy states, offering insights into the intricate interactions within adipose tissue during exercise.

In our flowcytometry analysis, we found significant increase in population of M2 (CD11c-/CD206+) ATMs from 21.0% to 34.8% and decrease in M1 ATMs (CD11c+/CD206-) from 37.6% to 29.3%., confirming the anti-inflammatory effect of exercise on macrophage polarization. A 37.6% prevalence of M1 ATMs in sedentary mice in our study may seem unusually high for a non-inflammatory model. However, it is crucial to contextualize these figures within the broader literature, where similar studies have reported inconsistent baseline populations of CD11c+ macrophages. For instance, Li et al. found that mice fed a high-fat diet (HFD) exhibited 31% CD11c+ M1 macrophages in epididymal adipose tissue, compared to only 3.6% in those on a normal diet (ND) after 20 weeks (19). Conversely, Kiran et al. reported 51.96% vs. 22.13% CD11c+ M1 macrophages in HFD and ND mice, respectively, after 12 weeks (20). These studies highlight that even within similar dietary conditions, baseline levels of CD11c+ M1 macrophages can vary significantly (3.6% vs. 22.13%), yet consistently show an increase with HFD. In our study, the mice in the sedentary group, acquired at 6 weeks old, continued their pre-existing sedentary conditions for an additional 10 weeks under our observation, which included a 1-week acclimatization followed by the period during which the exercise group underwent a 1-week adaptation and an 8-week aerobic training regimen. Of note, the inherent sedentary conditions of these mice, from their birth until the end of our study, may have mimicked a relatively inactive lifestyle, which could influence an inflammatory status. Furthermore, the specific tissues sampled (visceral adipose tissue as opposed to only epididymal adipose tissue in the cited studies) may also influence baseline levels of M1 macrophages. Despite these variabilities, the notable difference in the prevalence of M1 macrophages between the sedentary and exercise groups (37.6% vs. 29.3%) underscores the potential anti-inflammatory effects of exercise on ATMs. This finding supports our results within the established framework of ATM behavior in metabolic contexts, suggesting the exercise-induced modulation of inflammation.

GO analysis in our study highlighted that the targeted genes by various miRNAs are involved in biological processes induced by exercise in mouse ATMs. Microarray analysis revealed that the most affected functional categories of gene induced by exercise in ATMs includes DNA transcription, transport, cell differentiation, protein phosphorylation, protein transport, ion transport, actin cytoskeleton organization, and cell adhesion. This aligns with prior research emphasizing the crucial role of metabolism in determining the phenotype and function of macrophages, which involves DNA transcription, protein transport, and protein phosphorylation (4, 21). Furthermore, functions such as ion transport, actin cytoskeleton organization, and cell adhesion were identified as vital functional targets for infiltration and polarization of macrophage (21–23). Consequently, the regulation of ATMs polarization through an 8-week exercise regimen in our study could be linked to genes with these functions.

KEGG pathway analysis in our study suggested that significant pathways implicated in responding to exercise and CLGI include Wnt signaling pathway, PI3K-Akt signaling pathway, NF-kappa B signaling pathway, MAPK signaling pathway, mTOR signaling pathway, AMPK signaling pathway, Insulin signaling pathway. These pathways have been known to be related to macrophage polarization (24–27). For instance, Wnt signaling mediates TLR pathway triggering the release of pro-inflammatory cytokines that recruit and polarize ATMs, leading to metaflammation (28). The PI3K/Akt pathway orchestrates macrophage response to metabolic and inflammatory signals, crucial in adipocyte physiology and glucose homeostasis (29). Moreover, insulin and lipopolysaccharide mediated signaling in ATMs regulates postprandial glycemia through AKT-mTOR activation (30). Our data implied that the PI3K-Akt, MAPK, mTOR, AMPK, and Insulin signaling pathway may be the key regulatory mechanisms to investigate the adipose tissue in response to exercise.

The regulation of miRNAs is pivotal for the cellular inflammatory response in ATMs, particularly post-exercise. Our study identifies six miRNAs (miR-511-5p, miR-212-5p, miR-142-3p, miR-31-5p, miR-1894-3p, and miR-7b-5p) that correlate with macrophage polarization within ATMs following an exercise regimen. Notably, the upregulation of miR-511-5p and miR-212-5p could elucidate the anti-inflammatory and metabolic benefits of exercise. For instance, miR-511-5p has been implicated in glucocorticoid-mediated downregulation of TLR4 signaling activity, influencing macrophage polarization towards an M2 phenotype (31), and promoting M2 polarization while reducing inflammation in allergen-exposed mice (32). Similarly, the overexpression of miR-212-5p in macrophages has been associated with reduced triglyceride accumulation and an M2 phenotype enhancement in various models (33, 34). Conversely, the observed post-exercise downregulation of miR-142-3p, miR-31-5p, miR-1894-3p, and miR-7b-5p aligns with their known roles in inflammation and metabolic regulation. For example, miR-142-3p, typically elevated in obesity, has been linked to macrophage immunomodulation and was similarly decreased in our study, suggesting a shift towards an anti-inflammatory state (35–37). miR-31-5p is an inflammation-associated miRNA (38). Its suppression, shown to promote an M2 phenotype and reduce inflammation through AMPK/SIRT1/NLRP3 pathways (39), was also evident in our findings, reinforcing its potential therapeutic role in inflammation control. miR-1894-3p, known to be associated with inflammation and breast cancer (40), was not well studied in the context of macrophage polarization. Its downregulation in our exercised mouse mode suggests new pathways for future investigation. Additionally, the downregulation of miR-7b-5p observed in our study, was reported predominantly in M2 macrophages compared to M1 macrophages, further emphasizing the broad anti-inflammatory impact of exercise (41). These findings suggest that exercise not only improves metabolism but also promotes an anti-inflammatory macrophage polarization, potentially alleviating chronic low-grade inflammation (CLGI) in adipose tissue. The identified miRNAs serve as promising biomarkers for evaluating the effectiveness of exercise interventions in enhancing metabolic health. Future research should continue to explore how exercise modulates these specific miRNAs to fully understand their roles in metabolic and inflammatory pathways.

In this study, we observed that deregulation of the six miRNAs post-exercise affects genes involved in inflammatory and metabolic signaling pathways. Specifically, genes such as Fgf23 and Prkcb, which have been found to be highly expressed in individuals with metabolic syndrome (42, 43), are among the targets. Additionally, gene like Hras, Tnfrsf1a, Prkcb, and Wnt2b are associated with inflammation (44–47). The expression of Wnt2b, which is known to be upregulated by polarization-promoting factors in macrophages, contribute to the polarization of tumor-associated macrophages (45). Furthermore, Mapk10, Erbb3, Irs1, Nsf, and Tp53inp2 are genes linked with reduced adiposity and enhanced insulin sensitivity (48–52). MAPK10 (also known as JNK3) is noted for its protective role against excessive adiposity (51), while TP53INP2 serve as a negative regulator of adipogenesis (52). The insulin receptor substrate proteins IRS1, a key target of the insulin receptor tyrosine kinase, is often defective in tissues from insulin-resistant and diabetic individuals (49). Studies have also shown that exercise is associated with the expression of genes such as Erbb3, Irs1, Tnfrsf1a, Fgf23, and Hras (53–57). The ErbB receptors are involved in myogenesis and glucose transport in skeletal muscle, and exercise has been shown to activate ErbB3, and ErbB4 signaling (53). Exercise-induced increases in IRS-1 expression, a major marker of insulin signaling, are associated with improved insulin sensitivity (56). Notably, an one-year exercise intervention has led to a significant decrease in BMI and FGF-23 in overweight or obese children and adolescents (57). Given the limited understanding of the impact of exercise on Mapk10, Nsf, Tp53inp2, Prkcb, Wnt2b, future research should focus in functional analysis of these genes to elucidate their roles in exercise-related metabolic regulation.

This study is subject to several limitations that should be considered. Primarily, the ATMs sorted from mice may not fully represent human due to inherent species differences underscoring the need for validation through human studies. In addition, this study utilized solely male mice, hence further research is necessary to understand the gene expression in ATMs in female mice, which could differ significantly. Regarding food consumption, while this study did not directly monitor food intake, findings from another ongoing study using the same exercise protocol and animal model (unpublished data) suggest that exercise does not affect food consumption, isolating the effects of exercise from dietary influences. Furthermore, our investigation is restricted to examining the effects of an 8-week regimen of moderate-intensity aerobic exercise. Future research should expand to assess the impact of various types and intensities of exercise on ATMs. Importantly, although this study identified changes in six specific miRNAs associated with metabolic improvements post-exercise, the related gene targets and signaling pathways were predicted via computational methods. Rigorous experimental validations are essential to confirm these findings and clarify their implications in macrophage polarization and metabolic regulation.

In conclusion, our study elucidates the benefits of an 8-week moderate-intensity aerobic exercise regimen, demonstrating significant improvements in glucolipid metabolism and the promotion of an anti-inflammatory M2 macrophage phenotype in ATMs. This exercise regimen led to notable changes in miRNA expression within ATMs, highlighting the adaptability of immune cells to environmental cues such as physical activity. Specifically, 62 miRNAs were differentially expressed post-exercise, with six miRNAs showing consistent alterations that are potentially linked to enhanced metabolic health and immune regulation. These miRNAs and their computationally predicted gene targets are involved in crucial pathways that govern macrophage polarization and general metabolic processes, underscoring the broad regulatory capabilities of exercise on systemic health. These findings pave the way for further investigations into the mechanisms by which exercise can modulate immune and metabolic functions in non-pathological conditions, potentially guiding future interventions aimed at maintaining metabolic health and preventing chronic conditions through lifestyle modifications.

## Data Availability

The datasets presented in this study can be found in online repositories. The names of the repository/repositories and accession number(s) can be found below: GSE261688 (GEO).
