# Supplementary material for: The effects of exercise on microRNA expression profiling in adipose tissue macrophages of mice
Source: Front Immunol. 2024 Aug 19;15:1412621. doi: 10.3389/fimmu.2024.1412621 (PMC11366585; doi:10.3389/fimmu.2024.1412621)
Supplement: Supplementary file 1 [file Datasheet1.pdf]

frontiers

Supplementary Material

1 Supplementary Figures

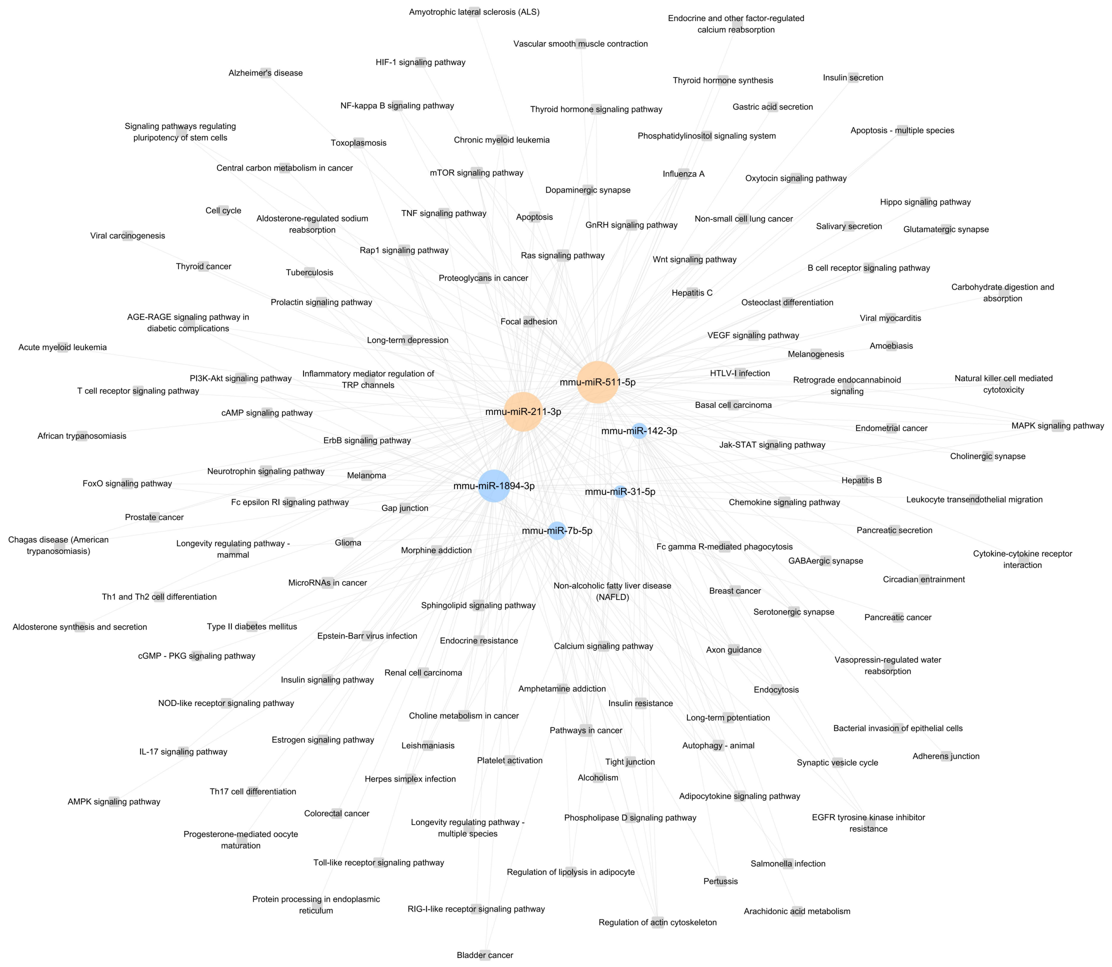

Supplemental Figure S1 The detailed miRNA-pathway-network, illustrating the relationships between the six miRNAs related to metabolic syndrome and their target genes.

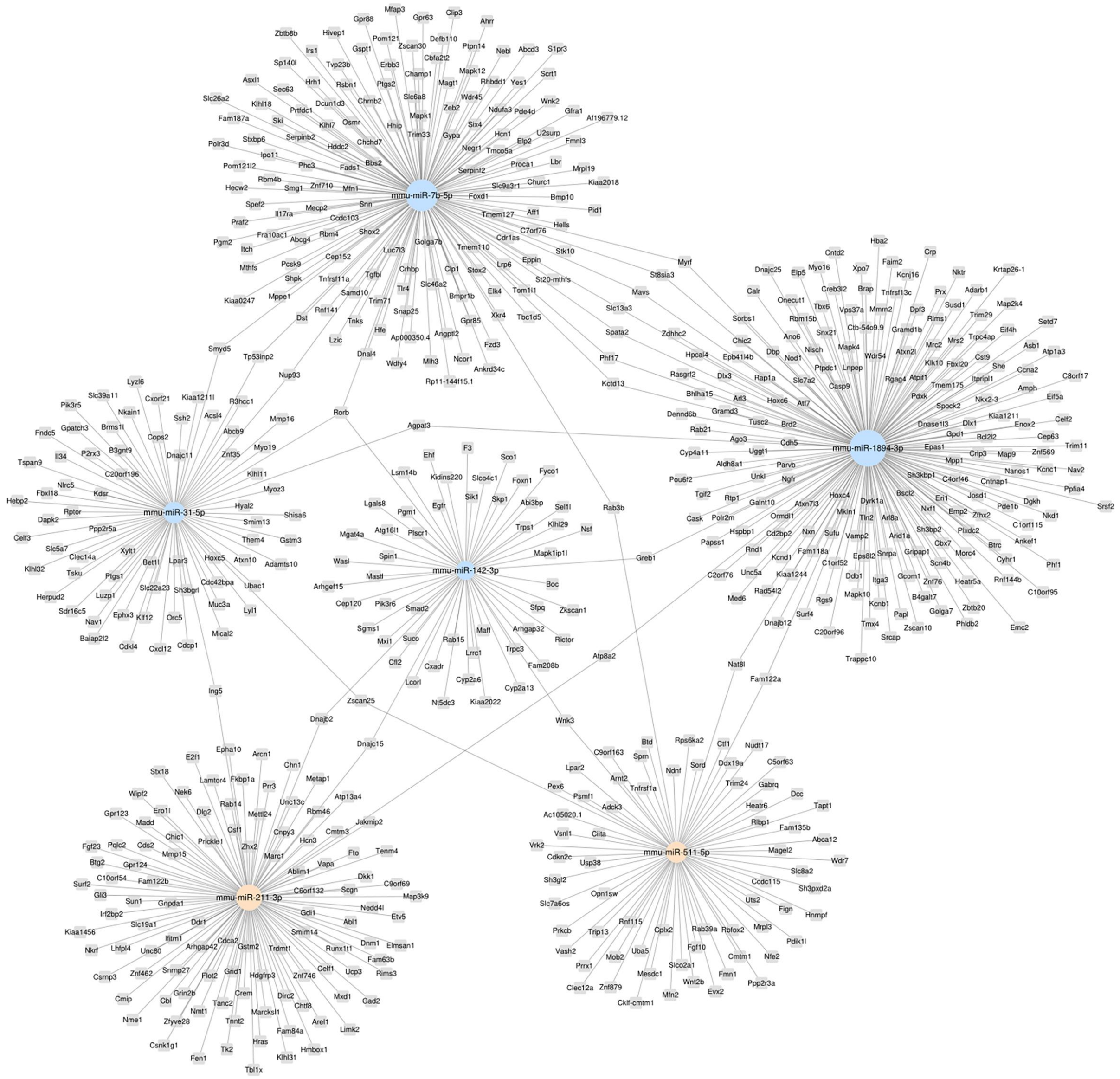

Supplemental Figure S2 the detailed miRNA-gene-network of the six miRNAs differentially expressed post-exercise**.**

2 Supplementary Tables

Table S1. Differentially expressed up-regulated miRNAs between sedentary and exercise groups in ATMs.

miRNA	Fold change	P value	Adj. P value
mmu-miR-7045-3p	1.84	0.001376	0.0247
mmu-miR-3620-3p	1.79	0.000269	0.0207
mmu-miR-669m-5p	1.558	0.001820	0.0247
mmu-miR-466m-5p	1.558	0.001820	0.0247
mmu-miR-345-5p	1.464	0.002665	0.0253
mmu-miR-211-3p	1.444	0.006518	0.0260
mmu-miR-511-5p	1.444	0.013076	0.0267
mmu-miR-6936-5p	1.444	0.016468	0.0267
mmu-miR-1955-3p	1.424	0.004118	0.0253
mmu-miR-149-3p	1.424	0.009471	0.0260
mmu-miR-3072-3p	1.395	0.005263	0.0260
mmu-miR-6538	1.395	0.015288	0.0267
mmu-miR-7003-3p	1.385	0.020514	0.0268
mmu-miR-302b-3p	1.366	0.001926	0.0247
mmu-miR-6923-3p	1.357	0.011461	0.0260
mmu-miR-212-5p	1.357	0.016655	0.0267
mmu-miR-3063-5p	1.357	0.019854	0.0268
mmu-miR-135a-1-3p	1.357	0.045263	0.0453
mmu-miR-6919-3p	1.347	0.004264	0.0253
mmu-miR-6236	1.347	0.007202	0.0260
mmu-miR-1894-5p	1.347	0.021942	0.0273
mmu-miR-99b-5p	1.329	0.031052	0.0347
mmu-miR-466h-5p	1.32	0.007621	0.0260
mmu-miR-127-3p	1.32	0.015366	0.0267
mmu-miR-362-3p	1.31	0.006701	0.0260
mmu-miR-6909-3p	1.31	0.008430	0.0260
mmu-miR-7686-5p	1.31	0.020793	0.0268
mmu-miR-3113-3p	1.31	0.031978	0.0352
mmu-let-7e-3p	1.301	0.009134	0.0260
mmu-miR-7238-3p	1.301	0.010256	0.0260
mmu-miR-6934-5p	1.301	0.023407	0.0282

Table S2. Differentially expressed down-regulated miRNAs between sedentary and exercise groups in ATMs.

miRNA	Fold change	P value	Adj. P value
mmu-miR-142-3p	-1.301	0.018189	0.0268
mmu-miR-7234-3p	-1.301	0.018413	0.0268
mmu-miR-344d-3p	-1.301	0.019637	0.0268
mmu-miR-471-3p	-1.301	0.025021	0.0296
mmu-miR-467g	-1.301	0.034496	0.0374
mmu-miR-669l-5p	-1.31	0.019870	0.0268
mmu-miR-6409	-1.31	0.029425	0.0338
mmu-miR-6983-5p	-1.31	0.042279	0.0434
mmu-miR-302c-3p	-1.32	0.022527	0.0275
mmu-miR-29c-5p	-1.32	0.030503	0.0345
mmu-miR-5619-3p	-1.32	0.041655	0.0433
mmu-miR-144-5p	-1.329	0.007902	0.0260
mmu-miR-3082-3p	-1.329	0.012824	0.0267
mmu-miR-1966-5p	-1.338	0.036616	0.0386
mmu-miR-7083-3p	-1.347	0.009365	0.0260
mmu-miR-7211-5p	-1.347	0.034988	0.0374
mmu-miR-669a-3-3p	-1.357	0.016793	0.0267
mmu-miR-6994-5p	-1.366	0.010905	0.0260
mmu-miR-1894-3p	-1.366	0.015374	0.0267
mmu-miR-23b-5p	-1.376	0.027711	0.0323
mmu-miR-7b-5p	-1.385	0.019572	0.0268
mmu-miR-8105	-1.385	0.019644	0.0268
mmu-miR-542-5p	-1.385	0.021795	0.0273
mmu-miR-3569-5p	-1.414	0.044216	0.0448
mmu-miR-3108-5p	-1.424	0.020848	0.0268
mmu-miR-1a-3p	-1.464	0.011385	0.0260
mmu-miR-31-5p	-1.505	0.003129	0.0253
mmu-miR-433-3p	-1.505	0.003546	0.0253
mmu-miR-7009-5p	-1.526	0.001115	0.0247
mmu-miR-6906-5p	-1.58	0.017850	0.0268
mmu-miR-5134-5p	-1.602	0.011996	0.0264
